# Hepatitis E in Thailand: From Seroprevalence to Foodborne and Transfusion-Associated Risks

**DOI:** 10.3390/jcm15082837

**Published:** 2026-04-09

**Authors:** Yong Poovorawan, Sitthichai Kanokudom, Pornjarim Nilyanimit, Jiratchaya Puenpa

**Affiliations:** 1 Center of Excellence in Clinical Virology, Faculty of Medicine, Chulalongkorn University, Bangkok 10330, Thailand; sitthichai.k@chula.ac.th (S.K.); mim_bhni@hotmail.com (P.N.); jiratchaya.ph@chula.ac.th (J.P.); 2 Fellow of the Royal Society of Thailand (FRS [T]), Sanam Sueapa, Dusit, Bangkok 10300, Thailand

**Keywords:** Hepatitis E virus (HEV), seroprevalence, adults, porcine, fecal-oral route, Thailand

## Abstract

**Background:** Hepatitis E virus (HEV) is an increasingly recognized cause of acute viral hepatitis in Thailand as the burden of hepatitis A, B, and C has declined. HEV is a positive-sense RNA virus in the family Hepeviridae with three major open reading frames encoding replication proteins (ORF1), the capsid protein (ORF2), and an accessory protein involved in viral egress (ORF3). Unlike highly endemic regions where genotypes 1 and 2 are linked to waterborne outbreaks, infections in Thailand are reported mainly as sporadic cases associated with zoonotic transmission, most commonly genotype 3. **Objectives:** This review summarizes the epidemiology, transmission routes, and public health implications of HEV infection in Thailand. **Methods:** Peer-reviewed studies on HEV seroprevalence, molecular epidemiology, and transmission in Thailand were identified through PubMed using combinations of the keywords “HEV” and “Thailand”. Two investigators independently screened titles, abstracts, and full texts. Eligible studies were synthesized qualitatively. **Results:** Earlier studies suggested low population exposure, but more recent evidence indicates substantial cumulative risk. A nationwide survey among blood donors reported anti-HEV IgG seroprevalence of about 30%, with geographic variation and increasing prevalence with age. Detection of HEV RNA in pigs, slaughterhouse environments, and retail pork products, together with links to raw or undercooked pork consumption, supports pigs as the principal reservoir and foodborne exposure as an important route. Transfusion-associated infection has also been documented. **Conclusions:** In Thailand, HEV infection is linked mainly to zoonotic and foodborne transmission involving genotype 3. Stronger surveillance, food safety measures, and risk-based blood safety policies are needed.

## 1. Introduction

Viral hepatitis remains a major global public health challenge and comprises five principal viruses: hepatitis A, B, C, D, and E. Among these, hepatitis B and C viruses are of particular concern because of their ability to cause chronic infection that can progress to liver cirrhosis and hepatocellular carcinoma. Globally, viral hepatitis is estimated to result in approximately 1.3 million deaths each year, and about 304 million people are living with chronic hepatitis B or C infection [1]. In Thailand, hepatitis A infection has markedly declined over the past decades, largely as a result of improved sanitation and socioeconomic development, and it is no longer considered a major public health concern [2]. Although an effective vaccine against hepatitis A is available, it has not been included in the national immunization program. In contrast, universal hepatitis B vaccination for newborns, implemented since 1992, has led to a substantial reduction in hepatitis B virus (HBV) infection, making Thailand one of the countries that has achieved notable success in HBV prevention and control [3]. For hepatitis C virus (HCV), despite the absence of a vaccine, highly effective direct-acting antiviral therapies can achieve a cure, and national treatment strategies have been implemented, exemplified by the micro-elimination program in Phetchabun province [4]. Hepatitis D virus (HDV) is not considered a significant problem in Thailand [5]. As the burden of hepatitis A, B, and C has declined, increasing attention has been directed toward the hepatitis E virus (HEV).

Historically, most attention has focused on hepatitis A, B, and C viruses. HEV was traditionally regarded as a disease primarily affecting developing countries with poor sanitation, particularly in regions such as Africa, South Asia, and Latin America [6]. However, as the incidence of other viral hepatitis infections has decreased, HEV has gained increasing recognition, including in developed countries where sporadic cases are now more frequently reported [7]. This shift has highlighted the growing clinical and public health importance of HEV infection.

HEV infection ranges from asymptomatic infection and self-limited acute hepatitis to severe disease in selected populations. In particular, HEV infection can lead to fulminant hepatitis or acute liver failure, particularly in pregnant women, among whom case fatality has been reported to be as high as 20–30%, especially during the later stages of pregnancy [8,9]. Transmission through blood transfusion has also emerged as an additional concern, particularly for high-risk recipients. Transfusion-transmitted HEV, predominantly associated with genotypes 3 and 4, has been reported in Europe and in parts of Asia, including China and Japan; however, available data from the UK suggest that this route accounts for <1% of all HEV infections [10,11,12].

Because of these differences, HEV should be evaluated within country-specific epidemiological contexts. Accumulating evidence in Thailand suggests that HEV transmission is more closely linked to zoonotic and foodborne exposure, particularly through swine reservoirs and pork consumption, than to large waterborne outbreaks [13,14,15,16]. At present, the available evidence remains fragmented across seroprevalence studies, molecular and genotype reports, foodborne investigations, and transfusion-related observations. To better understand the epidemiological situation, pinpoint important information gaps, and back up evidence-based control and prevention efforts, a thorough synthesis of these results is required.

This narrative review aims to synthesize the available evidence on HEV infection in Thailand, with emphasis on seroprevalence, transmission routes, circulating genotypes, and major sources of infection. By integrating the available data in the Thai context, this review seeks to provide a clearer overview of the epidemiology of HEV and to inform future research as well as prevention and control strategies.

## 2. Narrative Review Methodology

This study was conducted as a non-systematic narrative review to summarize the current knowledge on hepatitis E virus (HEV) infection in Thailand. To enhance transparency in reporting, the review was conducted in accordance with the Preferred Reporting Items for Systematic Reviews and Meta-Analyses extension for Scoping Reviews (PRISMA-ScR) checklist (Table S1). The review aimed to synthesize available evidence on HEV seroprevalence, transmission patterns, and key epidemiological characteristics in the Thai context, with particular emphasis on foodborne transmission and transfusion-associated infections, both of which are of clear public health relevance in Thailand.

**Figure 1:**
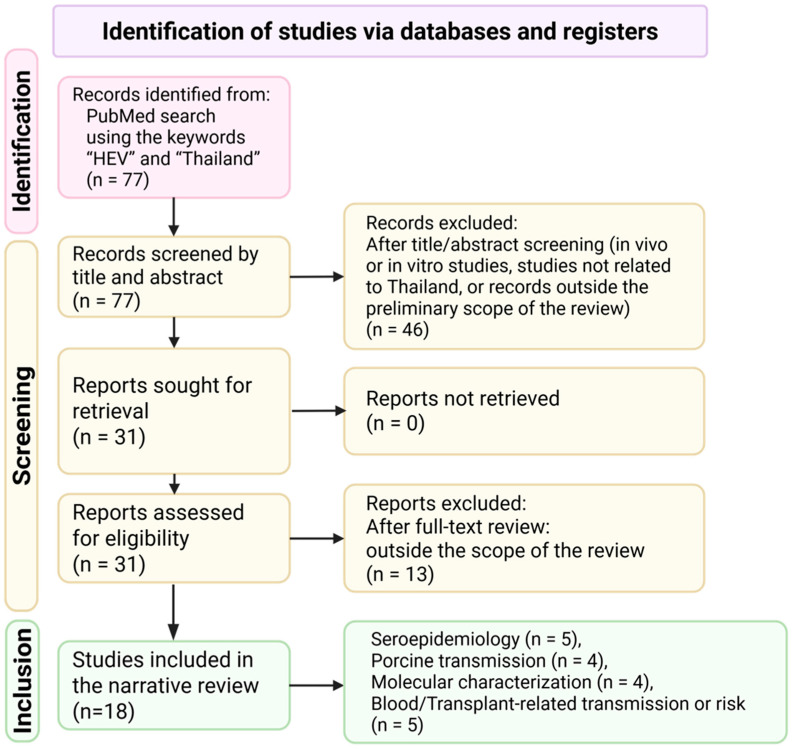
Flow diagram of literature search and study selection.

A literature search was performed in the PubMed database using the keywords “HEV” and “Thailand”. The search was conducted between January and March 2026 and yielded 77 records (Figure 1). Two investigators independently screened titles and abstracts to identify potentially relevant articles. After title and abstract screening, 46 records were excluded, and 31 full-text reports were sought for retrieval. All 31 reports were successfully retrieved and assessed independently in full by the two investigators.

When multiple publications were based on the same dataset or contained overlapping information, the less detailed and/or less original report was excluded in favor of the most informative publication. Any disagreements in study selection were resolved through discussion and consensus between the two investigators. Following full-text assessment, 13 reports were excluded because they were outside the predefined scope of the review, and 18 studies were ultimately included in the narrative review. The included evidence was synthesized qualitatively to summarize the burden of HEV infection in Thailand, circulating genotypes, and the predominant routes of transmission, with specific attention to foodborne exposure and transfusion-related transmission.

## 3. Virology

Hepatitis E virus (HEV) is a small, non-enveloped virus with an icosahedral capsid belonging to the family Hepeviridae, genus *Paslahepevirus* (previously *Orthohepvirus A*) [17]. The viral genome consists of a single-stranded, positive-sense RNA of approximately 7.2 kb, capped at the 5′ end and polyadenylated at the 3′ end. The genome contains three partially overlapping open reading frames (ORFs) that are essential for viral replication, assembly, and host interaction [18,19].

ORF1 encodes a large nonstructural polyprotein involved in viral replication and processing, comprising several functional domains, including methyltransferase, papain-like cysteine protease, helicase, and RNA-dependent RNA polymerase. ORF2 encodes the capsid protein, which plays a critical role in virion assembly, host cell attachment, and induction of the host immune response. ORF3 encodes a small multifunctional phosphoprotein that is involved in virion egress and modulation of host cellular pathways, particularly in quasi-enveloped HEV particles circulating in the bloodstream [18,20]. ORF2 and ORF3 are translated from a subgenomic RNA produced during viral replication [19]. HEV has been categorized as a non-enveloped virus, but it now exists in two forms: a feces-shedding form and a bloodstream-circulating quasi-enveloped form [21]. Biologists believe quasi-enveloped particles enter cells differently and may enable the virus elude host immune responses and expand beyond the liver [21,22].

## 4. Hepatitis E Genotypes and Classification

**Figure 2:**
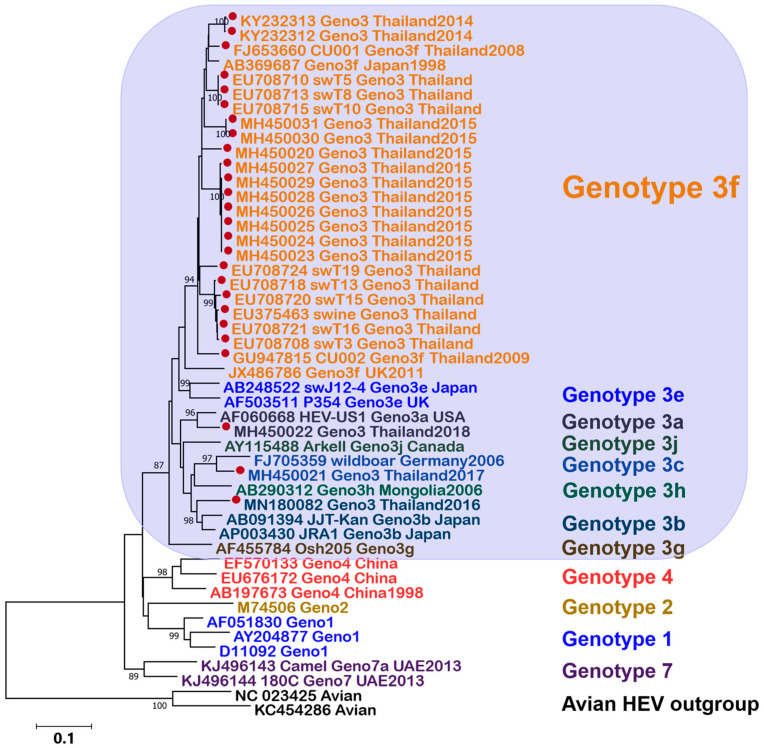
Maximum-likelihood phylogeny of hepatitis E virus (HEV) inferred from partial ORF2 sequences. The blue shaded area highlights all genotype 3 sequences, reflecting the exclusive detection of genotype 3 among Thai HEV strains. Thai sequences (retrieved from previously published studies and/or public databases; references provided) are indicated by solid red dots. Sequence labels are color-coded by genotype and subgenotype as shown in the colored text annotations on the right. The tree was reconstructed in MEGA X (version 10.2.6) [38] using the Tamura–Nei 1993 model with gamma-distributed among-site rate variation (TN93+G). Node support was assessed with 1000 bootstrap replicates, and values ≥80% are shown at the corresponding nodes. Genotypes are indicated by colored side bars. The tree is rooted with avian HEV outgroup sequences (NC_023425 and KC454286). The scale bar represents 0.1 substitutions per site.

Based on complete genome phylogenetic analyses, HEV within *Paslahepevirus* is classified into eight genotypes (HEV-1 to HEV-8) and 36 proposed subtypes [23]. Among these, genotypes 1–4, and occasionally genotype 7, have been reported in humans [24]. Genotypes 1 and 2 are human-restricted and are primarily transmitted via the fecal–oral route, frequently causing waterborne outbreaks in settings with limited sanitation [25,26]. HEV-1 is the predominant cause of outbreaks across South Asia, and has also been reported in parts of Africa and the Middle East, whereas HEV-2 shows a more focal distribution, historically linked to outbreaks in Mexico and later reported in several African countries, including Nigeria and Namibia [27,28,29]. In contrast, genotypes 3 and 4 are zoonotic, circulating in a wide range of animal hosts, particularly domestic pigs and wild boars, with human infection typically acquired through foodborne exposure, such as undercooked meat, or direct animal contact [30]. Genotype 3 predominates in Europe and other industrialized regions, including North America and Japan, whereas genotype 4 is reported mainly in Asia, particularly East Asia, including China [31,32]. Genotypes 5, 6, and 8 are considered animal-restricted, whereas genotype 7 has been reported in camels and has occasionally been associated with human infection [25,33]. HEV-7 sequences were first reported from three dromedaries sampled in the United Arab Emirates in 2013, and subsequent studies indicate that HEV-7 is geographically widespread in dromedaries [31]. Documented human HEV-7 infections remain rare, including a case of chronic infection in a liver transplant recipient [33]. In our phylogenetic analysis, Thai HEV reference sequences clustered predominantly within genotype 3 [34,35,36,37], with most grouping in a well-supported subtype 3f clade, while a smaller number clustered within other genotype 3 subgenotypes (Figure 2).

## 5. Disease Burden of Hepatitis E

HEV infection affects individuals across all age groups, although incidence patterns vary by viral genotype and geographic region [25]. Globally, HEV is estimated to cause approximately 20 million infections annually (based on 2005 estimates), including about 3.3 million symptomatic cases and tens of thousands of deaths each year. While many infections are asymptomatic or self-limited, HEV can cause severe disease, including acute liver failure and death, particularly among vulnerable populations.

The highest disease burden is observed in South Asia and parts of eastern sub-Saharan Africa, where genotypes 1 and 2 remain endemic and drive large waterborne outbreaks. In these high-endemic settings, incidence is often greatest among young adults aged 15–39 years [25]. Outbreaks in areas with inadequate water and sanitation infrastructure place substantial strain on healthcare systems and generate significant socioeconomic consequences, including hospitalizations and loss of productivity.

In contrast, in countries with relatively well-developed sanitation and healthcare infrastructure, such as Thailand, hepatitis E is more commonly detected as sporadic cases rather than large outbreaks. The circulating strains in Thailand are predominantly HEV genotype 3 [36,37,39,40,41], which is associated with zoonotic transmission and exhibits epidemiological patterns distinct from the waterborne genotypes prevalent in highly endemic regions. These genotype-specific differences contribute to regional variation in transmission dynamics and overall disease burden.

## 6. Hepatitis E Virus Infection in Thailand

In 1988, Thailand was reported to have a high prevalence of HEV infection, which at that time was broadly classified as non-A, non-B hepatitis [42]. This assumption was largely extrapolated from epidemic data reported in neighboring countries, including India and Myanmar, as well as Thailand itself. However, no direct empirical evidence or documented references were available to support this claim.

Subsequently, an enterically transmitted hepatitis virus distinct from hepatitis A and B, characterized by a relatively short incubation period and fecal–oral transmission, was identified and later designated as the hepatitis E virus [43]. In comparison with hepatitis A, both HAV and HEV are primarily transmitted through the fecal–oral route. The clinical manifestations of acute infection caused by these viruses are generally comparable, including symptoms such as fever and jaundice. However, acute hepatitis E is more commonly observed in older individuals and in those with underlying comorbidities, whereas acute hepatitis A is typically seen in children and younger adults [44]. Following the development of serological assays for detecting antibodies against HEV, seroprevalence studies were conducted across different population groups and age strata in Thailand. These studies consistently demonstrated a very low prevalence of anti-HEV antibodies in the Thai population [45]. Consequently, Thailand is not considered an endemic or hyperendemic area for hepatitis E.

## 7. Seroprevalence of Anti-HEV IgG in Thailand

Since the recognition of the HEV, serological studies assessing anti-HEV IgG prevalence in Thailand have been conducted for more than three decades. The earliest investigations, initiated around 1994 [45], examined diverse population groups, including children and adults, urban and rural residents, and blood donors. These early studies consistently demonstrated a very low prevalence of anti-HEV IgG, and Thailand was not considered an endemic area for HEV at that time.

A nationwide serosurvey among healthy Thai blood donors conducted in 2019 provided important updated evidence, revealing an overall anti-HEV IgG prevalence of 29.7% (*n* = 630) [46]. Marked regional differences were observed, with higher seroprevalence in the northern, northeastern, and central regions (approximately 29–36%) compared with the southern region (14.2%). The lower prevalence in southern Thailand may be explained by reduced swine density and distinct dietary practices that limit exposure to zoonotic HEV. Seroprevalence increased progressively with age, further supporting the concept of cumulative lifetime exposure [46,47].

Earlier studies among young Thai men also reported anti-HEV IgG seroprevalence of approximately 14%, with substantial provincial variation (3–26%), likely reflecting differences in environmental conditions, sanitation, and cultural behaviors such as pork consumption [48].

Clinical-based studies have provided additional insights into HEV exposure among specific patient populations. A cross-sectional laboratory-based study conducted at a tertiary-care hospital in Bangkok (2015–2016) reported anti-HEV IgG seropositivity among post–liver transplant recipients evaluated for suspected HEV infection, suggesting prior HEV exposure in this population [41]. This finding underscores the potential contribution of HEV infection to hepatic inflammation in clinical settings. Furthermore, among pediatric liver transplant recipients in Bangkok, anti-HEV IgG seroprevalence was approximately 15%, demonstrating that HEV exposure also occurs in pediatric and immunocompromised populations and may play a role in unexplained elevations of liver enzymes [48].

It should be noted that considerable heterogeneity exists among studies conducted in Thailand with respect to study period, diagnostic assays used, population characteristics, and geographic location. These methodological differences are likely to contribute to the wide range of reported seroprevalence estimates. A summary of key studies and their characteristics is presented in Table 1 [13,40,45,46,47,48,49,50].

## 8. Geographic Variation in HEV Seroprevalence in Thailand

A nationwide seroepidemiological survey conducted among Royal Thai Army conscripts, representing all provinces of Thailand, revealed significant geographic variation in exposure to HEV. The study also presented a heat map illustrating the provincial distribution of HEV seroprevalence across the country. Re-ported seroprevalence ranged from 3% to 26% across different provinces. The lowest seroprevalence was observed in southern Thailand, a region where a large proportion of the population practices Islam and dietary restrictions limit the consumption of pork [47].

## 9. Porcine Is the Possible Source of Infection

With advances in molecular epidemiology, diagnostic techniques for HEV detection and genotyping were developed. Subsequent investigations revealed sporadic cases of hepatitis E in Thailand, often associated with the consumption of pork products, such as undercooked pork dishes (e.g., Thai-style barbecue hotpot, also called in the Thai language “Moo Kratha”), or occupational exposure related to pig farming [49]. Surveillance studies detected HEV in pig sera and fecal samples, identifying genotype 3 strains, suggesting that pigs are a major reservoir for HEV in Thailand [14,34,37].

Indirect evidence supporting zoonotic transmission was demonstrated through seroepidemiological studies comparing populations with different dietary practices. In Narathiwat Province, located in southern Thailand and predominantly inhabited by Muslims who do not consume pork, anti-HEV antibodies were detected in fewer than 10% of adults. In contrast, approximately 50% of adults in Lopburi Province, where pork consumption is common, were seropositive for anti-HEV antibodies [13]. These findings strongly suggest that pigs play an important role in the transmission of HEV in Thailand.

Further investigations of pork products sold in fresh markets in Bangkok during 2014–2015, including 2205 samples of pork meat, pork liver, and intestines, detected HEV RNA in pork meat (0.36%) and pork liver (0.28%), whereas all intestinal samples tested negative. In parallel, among 1273 pig feces and bile samples collected from slaughterhouses, HEV RNA was identified with positivity rates of 5.4% and 2.92%, respectively [15]. Moreover, 540 pork products were collected from the vendors in Chiang Rai during 2020–2022. The findings showed that pork meat (1.30%), pork liver (2.59%), and pork intestine (0.74%) tested positive for HEV RNA [16]. Consumption of undercooked pork, handling raw pork during food preparation, and inadequate hand hygiene were recognized as significant risk factors for HEV infection. These findings support the classification of hepatitis E as a foodborne disease in Thailand.

## 10. HEV Can Be Transmitted via Blood Transfusion

In addition, hepatitis E cases have been reported among immunocompromised hosts and organ transplant recipients, including liver and kidney transplant patients, who frequently receive blood and blood products [52,53,54]. Blood transfusion has therefore been recognized as another potential route of HEV transmission. Screening of approximately 30,000 blood donations in 2015 detected HEV RNA in around 1 in 3300 Thai blood donations in Thailand [55]. These findings suggest that targeted HEV screening could be considered for blood products intended for high-risk recipients as a risk-reduction strategy in Thailand.

## 11. Severe Disease and Mortality in Vulnerable Groups

Although most HEV infections are self-limiting, certain populations are at significantly higher risk of severe disease and mortality. In developing countries experiencing HEV outbreaks, pregnant women are particularly vulnerable, with infection [56], predominantly caused by genotypes 1 and 2, associated with fulminant hepatic failure and markedly increased mortality compared with the general population. In contrast, in developed countries, severe HEV disease is more commonly observed among individuals with pre-existing chronic liver disease and immunocompromised hosts. These severe manifestations are most frequently linked to HEV genotypes 3 and 4, which differ in transmission patterns and clinical behavior from outbreak-associated genotypes [57].

## 12. Summary of Evidence and Implications

The available evidence indicates that HEV infection in Thailand is characterized mainly by sporadic, zoonotic, and foodborne transmission rather than by large waterborne outbreaks. Across the studies reviewed, genotype 3 appears to be the predominant lineage reported in Thailand. Consistent findings from seroprevalence studies, molecular investigations, and food surveys also support the view that swine serve as an important reservoir and that pork consumption is a major route of exposure. Geographic differences in seroprevalence, together with lower seropositivity in populations with limited pork consumption, further point to the importance of dietary and occupational exposure in the Thai setting. In addition, the detection of HEV RNA in blood donors suggests that transfusion-associated infection, although uncommon, may still be clinically relevant for high-risk recipients.

The reviewed evidence suggests that HEV in Thailand should not be viewed solely as an infectious disease issue, but also as a matter of food safety, blood safety, and One Health. The available literature further indicates that the burden of severe disease is concentrated in vulnerable groups, particularly immunocompromised individuals, transplant recipients, and pregnant women. These findings support prevention strategies that go beyond outbreak response alone and instead emphasize integrated surveillance, food safety measures, protection of high-risk groups, and evidence-based policy development.

## 13. Limitations of the Review

This review has several limitations. First, instead of a formal systematic or scoping review, it was carried out as a narrative review without a systematic approach. While the flow diagram and description of the literature search and study selection procedure were clear, it is important to note that the search was conducted solely in the PubMed database using a focused keyword strategy. Therefore, relevant studies available in other formats or indexed elsewhere may have been missed. Second, the narrative synthesis was based on only 18 studies, and the currently available Thai data are heterogeneous with regard to study populations, geographic contexts, and sampling periods. This heterogeneity restricts comparability across studies and prevents quantitative synthesis. Finally, there is limited data from nationally representative datasets; most of what we know about the prevalence and distribution of HEV infection in Thailand comes from studies that only included certain populations or regions.

## 14. National Policy for the Prevention of Hepatitis E in Thailand

HEV infection in Thailand occurs predominantly as sporadic cases related to zoonotic and foodborne transmission, with pigs serving as the main reservoir. Consumption of raw or undercooked pork products is considered an important and likely major route of human infection in Thailand [13,14,15]. Although large waterborne outbreaks are rare, HEV remains a public health concern among vulnerable populations, including immunocompromised individuals, organ transplant recipients, and patients receiving blood transfusions. Mother-to-child transmission has been documented. National prevention strategies should therefore adopt a risk-based One Health approach integrating human, animal, food safety, and environmental sectors.

Strengthened surveillance is central to HEV prevention. Integrated systems combining clinical surveillance of acute hepatitis with laboratory confirmation, including HEV serology and RNA testing, should be prioritized. Sentinel surveillance in high-risk populations and routine HEV monitoring in pigs, slaughterhouses, and pork products would improve early detection and risk assessment.

Risk communication and food safety measures are essential. Public education should emphasize the avoidance of raw or undercooked pork and promote safe food handling practices, while occupational health guidelines should target high-risk groups such as pig farmers and slaughterhouse workers.

Blood safety policies should continue targeted HEV screening for products intended for high-risk recipients, with periodic reassessment of donor prevalence to guide future screening strategies. Clinical guidelines should recommend HEV testing in immunocompromised patients with unexplained hepatitis and prompt recognition and management of chronic HEV infection.

An effective vaccine against HEV is currently available and has been implemented in selected settings, particularly in China [58,59,60]. The recombinant hepatitis E vaccine HEV239 (Hecolin^®^), developed by Xiamen Innovax Biotech (Xiamen, China), is currently the only licensed vaccine against hepatitis E. It has been licensed in China since 2011 and, outside China, in Pakistan since 2020 [61,62,63,64,65,66]. Therefore, country-specific assessments of disease burden, prevention strategies, and the cost-effectiveness of vaccination are important to determine the potential role of immunization programs [67]. In Thailand, HEV incidence is considered relatively low, and the vaccine has not yet been incorporated into the national immunization schedule. However, vaccination may be considered for selected high-risk groups pending further evidence on availability, feasibility, and economic value [68,69,70]. Continued evaluation of epidemiological trends, alongside sustained investment in sanitation, research, laboratory capacity, and policy integration within broader zoonotic and viral hepatitis control frameworks, remains essential to support informed decision-making and to guide potential future vaccine deployment in endemic or high-risk settings.

## 15. Conclusions

A national policy for hepatitis E prevention in Thailand should focus on food safety, zoonotic control, protection of high-risk populations, and targeted blood screening, rather than outbreak-driven interventions. Through a One Health approach and evidence-based policymaking [71], Thailand may strengthen HEV prevention and control, protect vulnerable populations, and support cost-effective public health strategies.

## 16. Future Directions

Future research should prioritize nationally representative studies to better define the burden, geographic distribution, and major transmission pathways of HEV infection in Thailand. A stronger One Health framework integrating surveillance across humans, swine, food products, and environmental sources will be important for clarifying zoonotic and foodborne transmission dynamics. Public health efforts should also continue to promote safe food practices, particularly avoidance of raw or undercooked pork products.

Additional studies are needed to better characterize the risk of transfusion-transmitted HEV in Thailand and to determine whether targeted blood donor screening would be justified for high-risk recipients, particularly immunocompromised patients. Further evaluation of HEV vaccination is also warranted, including its protective effectiveness against genotype 3 infection and the cost-effectiveness of vaccination strategies in selected at-risk populations.

## Figures and Tables

**Table 1:** Seroprevalence of anti-HEV IgG in the Thai population.

Sampling Year	Anti-HEV IgG + ve (%)	Sample Sizes	Population (Years)	Provinces	Test Company	Published Year [Ref]
1992–1994	9–22	1038	Blood donors (17–55), Pregnant women (16–42), Children (1–18), Liver disease (13–64)	Bangkok, Khon Kaen, Nakornrajsrima, and Nakornsrithamrat (four regions)	Genelabs *	1996 [45]
undefined	23.3 (16.7–27.9)	408	Swine farmers (18–55), Poultry farmers (18–63), Government officers (19–60)	Nakorn-Nayok and nearby provinces	Genelabs *	2009 [49]
undefined	11.5 (4–19.5)	381; 21 males	Nursing cadets (16–41)	Bangkok	EIA *	2009 [50]
2007–2008	14 (3–26)	7760	RTMA * (18–30)	National wide (four regions)	DIA.PRO *	2014 [47]
2008–2011	38.1	614	Acute hepatitis (1–90)	Bangkok	DIA.PRO *	2014 [40]
2014	37.3 in Lop Buri; 8.9 in Narathiwat (mainly Muslim)	721	Healthy (0.5–60)	Lop Buri and Narathiwat	Euroimm *	2015 [13]
2013	29.7 (7.5–47.5)	630	Blood donors (18–64)	Nationwide (four regions)	Euroimm *	2019 [46]
2015–2016	55.6	108	PLT *	Bangkok	WANTAI *	2020 [41]
2020–2022	15	101	PLT * (2–18)	Bangkok	Euroimm *	2023 [48]
2003–2020	24	67	PLT * (1–9)	Bangkok	Euroimm *	2024 [50]
2023–2024	13	200	PLHIV*	Chiang Mai	Euroimm *	2026 [51]

* Abbreviations: RTMA, Royal Thai Men’s Army; PLT, post-liver transplant patient; PLHIV, people living with HIV; Genelabs, Genelabs Diagnostics (Singapore); EIA, Enzyme like immunoassay (developed by the Walter Reed Army Institute of Research); DIA.PRO, DAI.PRO (Milan, Italy); Euroimm, Euroimmun (Lübeck, Germany); WANTAI, WANTAI Biological Pharmacy Enterprise (Beijing, China).

## Data Availability

All relevant data are included within the article and its references.

## Supplementary Materials

The following supporting information can be downloaded at: https://www.mdpi.com/article/10.3390/jcm15082837/s1, Table S1: Completed PRISMA-ScR Checklist.
