# Supplementary material for: Hepatitis E in Thailand: From Seroprevalence to Foodborne and Transfusion-Associated Risks
Source: J Clin Med. 2026 Apr 9;15(8):2837. doi: 10.3390/jcm15082837 (PMC13116988; doi:10.3390/jcm15082837)
Supplement: Supplementary file 1 [file jcm-15-02837-s001.zip › jcm-4188984-supplementary.pdf]

Table S1. Completed PRISMA-ScR Checklist.

Note: The manuscript is presented as a narrative review rather than a formal scoping review. The checklist below has been completed as faithfully as possible against the current manuscript, with not-applicable or partial reporting noted where relevant.

SECTION	ITEM	PRISMA-ScR CHECKLIST ITEM	REPORTED ON PAGE # / COMMENTS
**TITLE**			
Title	1	Identify the report as a scoping review.	Title page (p. 1). The manuscript is identified as a Review / narrative review, not as a scoping review.
**ABSTRACT**			
Structured summary	2	Provide a structured summary that includes (as applicable): background, objectives, eligibility criteria, sources of evidence, charting methods, results, and conclusions that relate to the review questions and objectives.	Abstract (p. 1). Structured summary provided: background, objectives, methods, results, and conclusions are reported. Eligibility criteria are only partly described.
**INTRODUCTION**			
Rationale	3	Describe the rationale for the review in the context of what is already known. Explain why the review questions/objectives lend themselves to a scoping review approach.	Introduction (pp. 1-2). Rationale is described in the context of HEV in Thailand; however, the manuscript is framed as a narrative review rather than a scoping review.
Objectives	4	Provide an explicit statement of the questions and objectives being addressed with reference to their key elements (e.g., population or participants, concepts, and context) or other relevant key elements used to conceptualize the review questions and/or objectives.	Introduction (p. 2). Objectives are explicitly stated.
**METHODS**			
Protocol and registration	5	Indicate whether a review protocol exists; state if and where it can be accessed (e.g., a Web address); and if available, provide registration information, including the registration number.	Not applicable. No protocol or registration is reported.
Eligibility criteria	6	Specify characteristics of the sources of evidence used as eligibility criteria (e.g., years considered, language, and publication status), and provide a rationale.	Methods (p. 2). Partly reported: studies within the predefined Thai HEV scope were eligible; overlapping and out-of-scope reports were excluded. Years, language, and publication status were not explicitly specified.
Information sources*	7	Describe all information sources in the search (e.g., databases with dates of coverage and contact with authors to identify additional sources), as well as the date the most recent search was executed.	Methods (p. 2). PubMed was the only information source. The search was conducted between January and March 2026.
Search	8	Present the full electronic search strategy for at least 1 database, including any limits used, such that it could be repeated.	Methods (p. 2). Keywords ("HEV" and "Thailand") are reported, but the full reproducible electronic search strategy and limits are not provided.
Selection of sources of evidence†	9	State the process for selecting sources of evidence (i.e., screening and eligibility) included in the scoping review.	Methods and Figure 1 (pp. 2-3). Two investigators independently screened titles, abstracts, and full texts; disagreements were resolved by discussion and consensus.
Data charting process‡	10	Describe the methods of charting data from the included sources of evidence (e.g., calibrated forms or forms that have been tested by the team before their use, and whether data charting was done independently or in duplicate) and any processes for obtaining and confirming data from investigators.	Not explicitly described.
Data items	11	List and define all variables for which data were sought and any assumptions and simplifications made.	Partly described in the Methods and Objectives (p. 2): seroprevalence, transmission routes/patterns, circulating genotypes, burden, and public-health relevance.
Critical appraisal of individual sources of evidence§	12	If done, provide a rationale for conducting a critical appraisal of included sources of evidence; describe the methods used and how this information was used in any data synthesis (if appropriate).	Not done / not reported.

SECTION	ITEM	PRISMA-ScR CHECKLIST ITEM	REPORTED ON PAGE # / COMMENTS
Synthesis of results	13	Describe the methods of handling and summarizing the data that were charted.	Methods (p. 2). Included evidence was synthesized qualitatively.
**RESULTS**			
Selection of sources of evidence	14	Give numbers of sources of evidence screened, assessed for eligibility, and included in the review, with reasons for exclusions at each stage, ideally using a flow diagram.	Methods and Figure 1 (pp. 2-3). Numbers screened, assessed for eligibility, excluded, and included are reported, with reasons for exclusion.
Characteristics of sources of evidence	15	For each source of evidence, present characteristics for which data were charted and provide the citations.	Table 1 and Sections 4-10 (pp. 4-8). Characteristics of the included Thai evidence and corresponding citations are presented.
Critical appraisal within sources of evidence	16	If done, present data on critical appraisal of included sources of evidence (see item 12).	Not applicable. No formal critical appraisal was conducted.
Results of individual sources of evidence	17	For each included source of evidence, present the relevant data that were charted that relate to the review questions and objectives.	Sections 4-11 and Table 1 (pp. 4-9). Relevant results from individual included studies are presented narratively.
Synthesis of results	18	Summarize and/or present the charting results as they relate to the review questions and objectives.	Sections 12 and 15-16 (pp. 9-11). Results are summarized in relation to the review objectives, implications, conclusions, and future directions.
**DISCUSSION**			
Summary of evidence	19	Summarize the main results (including an overview of concepts, themes, and types of evidence available), link to the review questions and objectives, and consider the relevance to key groups.	Section 12, Summary of Evidence and Implications (p. 9). Main findings, themes, and relevance to key groups are summarized.
Limitations	20	Discuss the limitations of the scoping review process.	Section 13, Limitations of the Review (pp. 9-10).
Conclusions	21	Provide a general interpretation of the results with respect to the review questions and objectives, as well as potential implications and/or next steps.	Section 15, Conclusion and Section 16, Future Directions (pp. 10-11).
**FUNDING**			
Funding	22	Describe sources of funding for the included sources of evidence, as well as sources of funding for the scoping review. Describe the role of the funders of the scoping review.	Funding statement (p. 11). Sources of funding for the review and the role of the funders are described.
